# Association between contralateral adrenal and hypothalamus-pituitary-adrenal axis in benign adrenocortical tumors

**DOI:** 10.3389/fendo.2023.1199875

**Published:** 2023-07-25

**Authors:** Hongyuan Zhu, Luming Wu, Tingwei Su, Lei Jiang, Weiwei Zhou, Yiran Jiang, Cui Zhang, Xu Zhong, Weiqing Wang

**Affiliations:** ^1^ Shanghai Key Laboratory for Endocrine Tumors, Shanghai Clinical Centre for Endocrine and Metabolic Diseases, Shanghai Institute of Endocrine and Metabolic Diseases, Ruijin Hospital, Shanghai Jiaotong University School of Medicine, Shanghai, China; ^2^ Laboratory for Endocrine and Metabolic Diseases of Institute of Health Science, Shanghai Jiaotong University School of Medicine and Shanghai Institutes for Biological Sciences, Chinese Academy of Sciences, Shanghai, China

**Keywords:** contralateral adrenal, nonfunctional adrenocortical tumor, mild autonomous cortisol secretion, hypothalamus-pituitary-adrenal (HPA) axis, causal mediation analysis

## Abstract

**Context:**

Adrenal incidentaloma (AI) is commonly discovered on cross-sectional imaging. Mild autonomous cortisol secretion is the most common functional disorder detected in AI.

**Objective:**

To delineate the association between radiological characteristics of benign adrenocortical tumors and hypothalamus-pituitary-adrenal (HPA) axis.

**Methods:**

In the study, 494 patients diagnosed with benign unilateral adrenocortical tumors were included. Mild autonomous cortisol secretion (MACS) was diagnosed when cortisol after 1mg-dexamethasone suppression test (1-mg DST) was in the range of 1.8-5ug/dl. Non-functional adrenocortical tumor (NFAT) was diagnosed as cortisol following 1-mg DST less than 1.8ug/dL. We performed Logistics regression and causal mediation analyses, looking for associations between radiological characteristics and the HPA axis.

**Results:**

Of 494 patients, 352 (71.3%) with NFAT and 142 (28.7%) with MACS were included. Patients with MACS had a higher tumor diameter, thinner contralateral adrenal gland, and lower plasma ACTH and serum DHEAS than those with NFAT. ACTH (OR 0.978, 0.962-0.993) and tumor diameter (OR 1.857, 95%CI, 1.357-2.540) were independent factors associated with decreased serum DHEAS (all P<0.05). ACTH was also associated with decreased contralateral adrenal diameter significantly (OR 0.973, 95%CI, 0.957-0.988, P=0.001). Causal mediation analysis showed ACTH mediated the effect significantly for the association between 1-mg DST results and DHEAS level (P_mediation<_0.001, proportion=22.3%). Meanwhile, we found ACTH mediated 39.7% of the effects of 1-mg DST on contralateral adrenal diameter (P_mediation_=0.012).

**Conclusions:**

Patients with MACS had thinner contralateral adrenal glands and disturbed HPA axes compared with NFAT. ACTH may partially be involved in mediating the mild autonomous cortisol secretion to DHEAS and the contralateral adrenal gland.

## Introduction

With the development and widespread use of advanced technology, incidentally discovered adrenal tumors have become common in clinical practice. The prevalence of adrenal incidentalomas (AIs) has been reported to be 0.4% to 7.3% among adults, and it is higher in the elderly, reaching nearly 10% (1–4).

Most AIs are benign and asymptomatic and are often considered non-functional tumors. However, they could actually produce cortisol in amounts insufficient for leading to clinically apparent symptoms, known as mild autonomous cortisol secretion (MACS). According to the updated AI guidelines of the European Society of Endocrinology (5), MACS is defined as failure to suppress serum cortisol sufficiently after a 1-mg overnight dexamethasone suppression test (1-mg DST) in the absence of the classical signs or symptoms of overt Cushing’s syndrome. A post-dexamethasone cortisol level ≤1.8 µg/dL is considered non-functional, and levels above1.8 µg/dL indicate MACS. This mild cortisol excess is associated with adverse cardiometabolic consequences and related mortality (6–12).

There have been few studies on the relationship between radiological features of the adrenal adenomas or adrenal glands and the hypothalamus-pituitary-adrenal (HPA) axis (13–17). Although the relationship between radiological characteristics and cortisol secretion has been previously described, these studies did not evaluate the association between radiological features and the HPA axis, especially in patients with MACS (18, 19).

In this study, we aim to describe the clinical and radiological characteristics of patients with MACS and NFAT. To further assess the association, we performed logistic regression and causal mediation analysis.

## Materials and methods

### Patients

**Figure 1:**
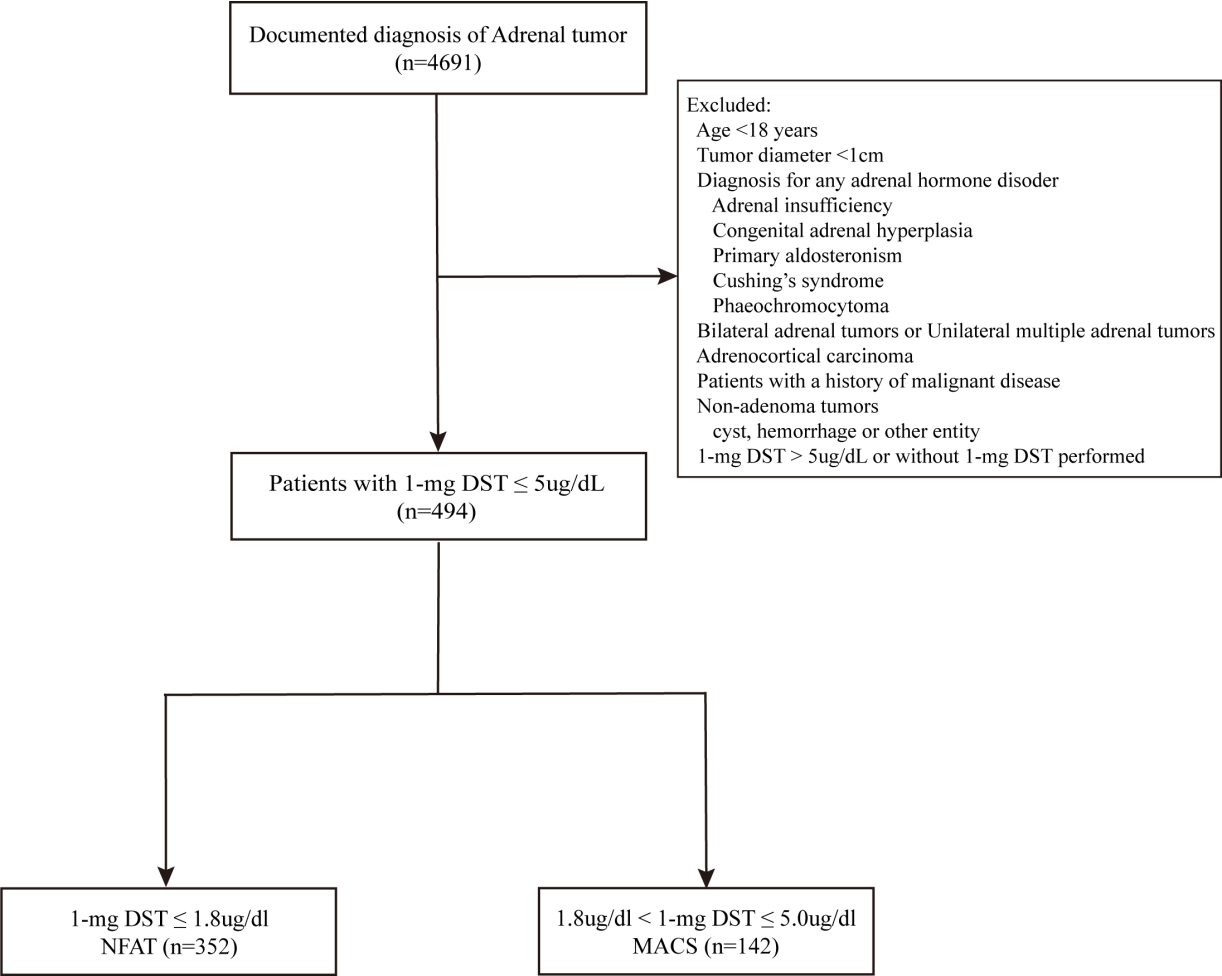
Flowchart of the study. 1-mg DST, 1-mg overnight dexamethasone-suppression test; NFAT, nonfunctional adrenocortical tumor; MACS, mild autonomous cortisol secretion.

This was a retrospective observational study. Patients with adrenal tumors were consecutively treated in the Department of Endocrine and Metabolic Diseases, Ruijin Hospital, from 2010 to 2020. All patients undergo a comprehensive evaluation aimed at assessing the functional status and potential malignancy of the adrenal lesion.

(**Supplementary Figure 1**). We selected 494 patients on the basis of the following exclusion criteria: (i) age younger than 18 years or adrenal tumor diameter <1cm; (ii) any adrenal hormone disorder, including adrenal insufficiency, congenital adrenal hyperplasia, primary aldosteronism, Cushing’s syndrome, and phaeochromocytoma; (iii) bilateral or unilateral multiple adrenal tumors; (iv) adrenocortical carcinoma: (v) patients with a history of malignant disease; (vi) non-adenoma tumors; (vii) 1-mg DST>5ug/dl. Patients who were using any medications known to affect steroid synthesis or metabolism, as well as those with pre-existing chronic kidney disease and liver cirrhosis, were also excluded prior to the administration of the 1-mg DST. Benign adrenocortical tumor was diagnosed using attenuation criteria <10 Hounsfield units (HU) in unenhanced computed tomography (CT) or stringent washout criteria when delayed contrast CT was available (absolute washout > 60% and/or relative washout > 40%). Finally, 494 patients with diagnosed benign adrenocortical tumors that had a 1-mg DST less than 5ug/dL were included (**Figure 1**). According to the latest updated guidelines (5), NFAT (n=352) was defined as cortisol following 1-mg DST less than 1.8ug/dL and MACS (n=142) was defined as cortisol following 1-mg DST in the range of 1.8-5.0ug/dL.

All patients gave their informed consent for participation. This study was approved by the ethics committee of Ruijin Hospital, Shanghai Jiao Tong University School of Medicine.

### Endocrine hormone assessments

All blood samples were detected in a College of American Pathologists (NO. 7217913)-accredited laboratory. We performed a 1mg overnight dexamethasone suppression test (DST) by administering 1mg of dexamethasone at 24:00, with measurement of cortisol the following morning (between 08:00 and 09:00 hours). Serum cortisol and 24h-UFC were measured by using an Access Immunoassay System (A Beckman Coulter Corp, Fullerton, CA, USA). Plasma ACTH levels were measured by using an ELSA-ACTH immunoradiometric assay method (Cisbio Bioassays, Codolet, France). The intraassay and interassay coefficients of variation were 6.1% and 5.3% for ACTH. The normal range for ACTH is 12-78 pg/ml. Serum-free testosterone (T) and androstenedione (AD) were measured by radioimmunoassay. Other sex hormones, such as dehydroepiandrosterone sulfate (DHEAS), SHBG, and T were measured by using a chemiluminescence immunoassay (Abbott Laboratories, Abbott Park, IL).

### Measurements of radiologic characteristics

Radiologic characteristics of the adrenal tumor and contralateral adrenal were evaluated in patients with unenhanced or pre-contrast CT images. The widths of the contralateral adrenal limb were defined as the mean of the maximum width of the limbs perpendicular to the long axis (medial and lateral) (18, 20). The diameters of the tumor were measured as the maximum width of the tumor. The CT scans were assessed by two different investigators who were not given information about the hormonal results. If there was a disagreement on measurements, investigators would re-evaluate the CT scans together and measure them again. All measurements were performed with a standard window setting for abdominal CT scans at Ruijin Hospital (width: 200 HU, level: 40 HU).

### Statistical analysis

Continuous variables were presented as the medians (interquartile ranges, 25%-75%), and categorical variables were displayed as percentages. Variables were tested for normal distribution using the Kolmogorov-Smirnov test. Normally distributed variables were analyzed by One-way ANOVA, and non-normal distributions variables were analyzed by the Mann-Whitney U test. Categorical variables were analyzed by the Chi-square test. A logistic regression model was performed with unadjusted and adjusted models (age and sex) assessing odds ratios for the variables associated with decreased DHEAS and contralateral diameter.

Causal mediation analysis was performed by using the R package mediation (version 4.5.0). Consistent with the previous study (21), relationship groups that met the following criteria were defined as significant causal relationships: (1) total effect is significant (P_total_ effect<0.05), (2) mediated proportion>10%, and (3) indirect effect is significant (P_mediation_<0.05). Moreover, we performed a sensitivity analysis using multiple imputation techniques to address missing data.

Statistical analysis was performed using SPSS (v.24: SPSS, Inc., Chicago, IL) and R 4.1.3 software. A two-sided P value < 0.05 was considered statistically significant.

## Results

### Clinical and endocrine characteristics

A total of 494 patients diagnosed with unilateral benign adrenocortical tumor were included in the study. According to the morning cortisol following 1-mg DST, 352 (71.3%) patients had nonfunctional adrenocortical tumors (NFAT), and 142 (28.7%) had mild autonomous cortisol secretion (MACS) and lacked the distinctive clinical features of overt cortisol excess. Women represented 54.0% of the patients included in the study, and the female predominance was mainly pronounced in MACS (65.5%) (**Table 1**). Patients with MACS were older than those with NFAT at the time of adrenal tumor diagnosis (57 years vs 55 years, p=0.004).

**Table 1:** Clinical and radiological characteristics of included patients.

Characteristic	NFAT (n=352)	MACS (n=142)	P value
Gender (male/female)	178/174	49/93	0.003
Age (years)	55 (45, 61)	57 (49, 65)	0.004
BMI (Kg/m^2^)	25.10 (23.00, 26.95)	24.49 (21.79, 27.65)	0.794
Serum cortisol (8am) (ug/dl)	11.24 (8.61, 14.43)	12.23 (9.74, 15.81)	0.014
Serum cortisol (0am) (ug/dl)	2.89 (1.93, 4.76)	3.99 (2.86, 5.44)	<0.001
ACTH (pg/ml)	25.30 (19.09, 37.80)	18.19 (13.77, 28.65)	<0.001
24h-UFC (ug/24h)	85.68 (65.69, 115.39)	92.40 (67.46, 119.05)	0.142
Cortisol after 1-mg DST (ug/dl)	1.08 (0.85, 1.37)	2.37 (2.01, 2.94)	<0.001
DHEAS (ug/dl)	140.00 (86.35, 224.45)	93.20 (58.80, 127.40)	<0.001
DHEAS Ratio	1.61 (1.01, 2.33)	1.13 (0.71, 1.90)	0.009
SHBG (nmol/L)	32.60 (22.73, 46.33)	35.50 (25.83, 53.58)	0.018
AD (ng/mL)	1.21 (0.92, 1.57)	1.07 (0.86, 1.43)	0.171
T (ng/mL)	0.87 (0.23, 4.05)	0.32 (0.19, 3.84)	0.041
FT (pg/ml)	3.75 (1.33, 8.49)	1.67 (1.09, 7.04)	0.003
DHT (pg/ml)	70.67 (30.78, 219.68)	45.89 (19.63, 133.41)	0.084
CT attenuation value (HU)	9.8 (-1.7, 20.1)	11.1 (-1.4, 21.7)	0.560
Tumor diameter (mm)	20.30 (15.65, 25.95)	23.25 (17.40, 30.23)	<0.001
Contralateral diameter (mm)	3.45 (3.00, 4.05)	3.20 (2.74, 3.63)	0.001
Diabetes (%)	21.93%	17.89%	0.344
Hypertension (%)	55.46%	62.79%	0.150
Dyslipidemia (%)	13.65%	14.29%	0.860

Data were medians (interquartile ranges) for continuous variables or proportions for categorical variables.

Serum cortisol (8am, 0am) and SHBG were significantly higher in MACS. In contrast, MACS had lower levels of plasma ACTH and androgens (serum DHEAS, T, and FT). To adjust for the potential impact of age and gender on DHEAS level, we utilized the DHEAS ratio and found that it was significantly decreased in the MACS group. Meanwhile, we found MACS had higher tumor diameter and thinner contralateral adrenal gland diameter than NFAT. However, no difference was observed for 24h-UFC between the two groups. In addition, the prevalence of cardiometabolic disease (including diabetes, hypertension, and dyslipidemia) did not differ between patients with NFAT and those with MACS.

We further presented the clinical and endocrine characteristics of different gender groups (**Supplementary Table 1**). Consistent with the total patients, MACS showed similar HPA axis changes in both groups. However, males showed significantly distinct radiological features; there was a similar trend in the female group but without statistically significant differences.

### Independent factors associated with decreased DHEAS and contralateral diameters

**Figure 2:**
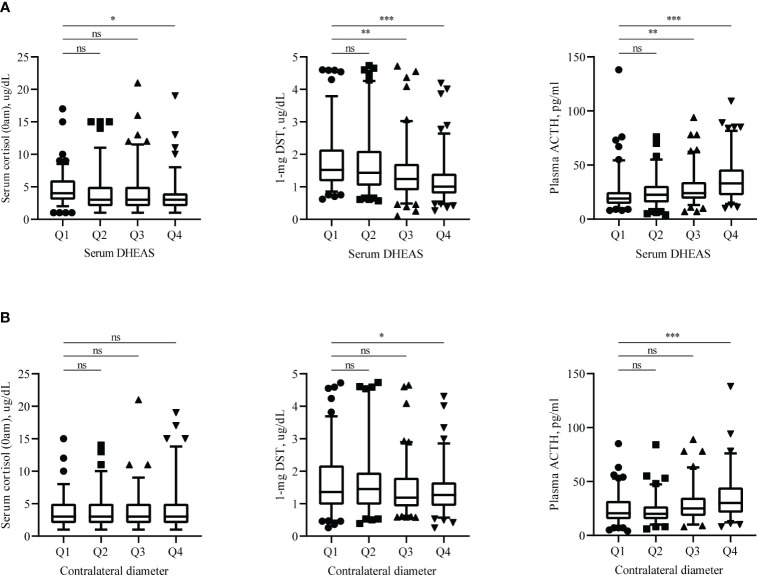
Endocrine assessments results. Distribution of serum cortisol (0am), plasma ACTH and 1-mg DST according to serum DHEAS **(A)** and contralateral adrenal diameter **(B)**. Endocrine hormones measured in these patients are shown as scatters, with boxes representing median and 5%-95% range. DHEAS = Dehydroepiandrosterone sulfate; 1-mg DST, 1mg overnight dexamethasone-suppression test. Q1: 0-25%, Q2: 25-50%, Q3: 50-75%, Q4:75-100%. ns: not statistically significant (P >0.05), *P < 0.05, **P < 0.01, and ***P < 0.001.

Significantly lower serum DHEAS and contralateral diameters were observed in patients with MACS. Grouping according to the quartiles, we found 1-mg DST and plasma ACTH may be associated with serum DHEAS and contralateral diameter (**Figure 2**).

To find the independent factors, we redivided the total patients into two new groups by the median of the serum DHEAS level or contralateral diameter and constructed logistic regression models [odds ratio (OR) and 95%CI]. In the multivariable logistic regression model adjusted for age and gender, we found tumor diameter (per 1-cm increase, OR 1.857 (1.357-2.540), P<0.001) was negatively associated and plasma ACTH was positively associated with decreased DHEAS, with OR (per 1-unit increase, 0.978 (0.962-0.993), P=0.006) (**Table 2**). About the decreased contralateral diameter, the univariable analysis showed that plasma ACTH and 1-mg DST were significantly associated with decreased contralateral diameter (**Table 3**). However, only ACTH was significantly associated with decreased contralateral diameter in multivariable logistic regression model (per 1-unit increase, OR 0.973 (0.957-0.988), P=0.001).

**Table 2:** Logistic regression analysis of decreased DHEAS.

	Decreased DHEAS			
	Univariable		Multivariable	
	OR (95% CI)	P value	OR (95% CI)	P value
ACTH	0.988 (0.978-0.998)	0.022	0.978 (0.962-0.993)	0.006
Serum cortisol (8am)	0.973 (0.933-1.015)	0.207	–	–
Serum cortisol (0am)	1.052 (0.984-1.125)	0.139	–	–
Cortisol after 1mg DST	1.513 (1.201-1.904)	<0.001	1.226 (0.923-1.628)	0.160
Tumor diameter	1.855 (1.398-2.462)	<0.001	1.857 (1.357-2.540)	<0.001

Odds ratio (OR) and 95% confidence interval (CI) was evaluated using a logistic regression model; a multivariable model was adjusted for age and gender.

**Table 3:** Logistic regression analysis of decreased contralateral diameter.

	Decreased contralateral diameter			
	Univariable		Multivariable	
	OR (95% CI)	P value	OR (95% CI)	P value
ACTH	0.966 (0.952-0.980)	<0.001	0.973 (0.957-0.988)	0.001
Serum cortisol (8am)	0.962 (0.921-1.005)	0.081	0.984 (0.937-1.034)	0.528
Serum cortisol (0am)	0.952 (0.886-1.024)	0.188	–	–
Cortisol after 1mg DST	1.418 (1.124-1.787)	0.003	1.258 (0.963-1.644)	0.092
Tumor diameter	1.114 (0.906-1.369)	0.305	–	–

Odds ratio (OR) and 95% confidence interval (CI) was evaluated using logistic regression models; multivariable models were adjusted for age and gender.

In the subgroup analysis of MACS group, we found plasma ACTH was associated with decreased DHEAS in multivariable logistic regression model, with OR (per 1-unit increase, 0.937 (0.888-0.988), P=0.016) (**Supplementary Table 2**). However, there was no significant variable associated with decreased contralateral diameter (**Supplementary Table 3**).

### Causal mediation analysis between radiological characteristics and endocrine assessments

**Figure 3:**
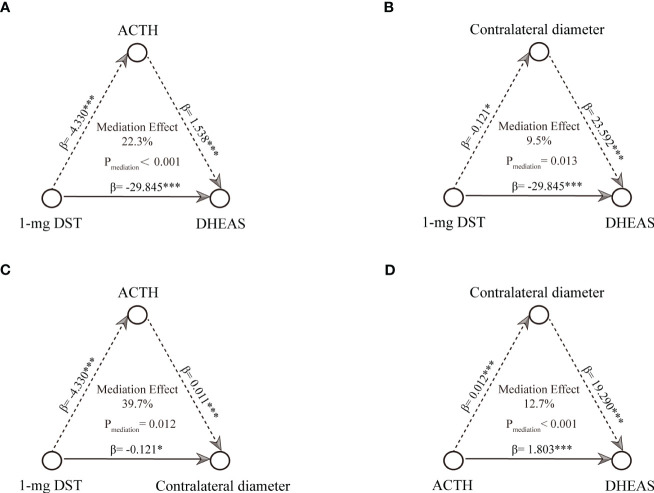
Causal mediation analysis among radiological characteristics, 1-mg DST and endocrine assessments. **(A)** mediation linkages between ACTH and 1-mg DST contributed DHEAS. **(B)** mediation linkages between contralateral diameter and 1-mg DST contributed DHEAS. **(C)** mediation linkages between ACTH and 1-mg DST contributed contralateral diameter. **(D)** mediation linkages between contralateral diameter and ACTH contributed DHEAS. *P < 0.05, **P < 0.01, and ***P < 0.001.

We first performed the causal mediation analysis on the linkages between tumor diameter, cortisol (including cortisol (8am, and 0am), and morning cortisol after the 1-mg DST) and ACTH. Significant causal mediation linkages of 1-mg DST were observed (P_mediation=_0.042, proportion=24.2%, **Supplementary Table 4**). To further investigate the concrete relationship, a causal mediation analysis between 1-mg DST and DHEAS was performed. We found that ACTH mediated the effects significantly (P_mediation_ <0.001, proportion=22.3%, **Figure 3A**), and contralateral adrenal diameter mediated 9.5% of the effects (P_mediation_ =0.013, **Figure 3B**). The plasma ACTH mediated 39.7% of the effects of 1-mg DST on contralateral adrenal diameter (P_mediation_ =0.012, **Figure 3C**). The contralateral adrenal diameter was shown to mediate the effects of ACTH on DHEAS significantly (P_mediation_ <0.001, proportion=12.7%, **Figure 3D**). Additionally, the sensitive analysis showed ACTH mediated 16.2% of the effects between 1-mg DST and DHEAS and 34.0% of the effects between 1-mg DST and contralateral adrenal diameter (both P_mediation_ <0.05, **Supplementary Figures 2A, C**). The contralateral adrenal diameter mediated 10.9% of the effects between 1-mg DST and DHEAS and 18.3% of the effects between ACTH and DHEAS (**Supplementary Figures 2B, D**).

## Discussion

In the present study, we found patients with MACS had a lower serum DHEAS and contralateral diameter than those with NFAT. Multivariable logistic regression models found ACTH and tumor diameter were independent factors associated with decreased serum DHEAS levels. For the decreased contralateral diameter, ACTH was significantly associated. Furthermore, significant mediation linkages for association of 1-mg DST and DHEAS through ACTH were observed, which is consistent with the hypothesis that low DHEAS levels can reflect chronically suppressed HPA axis (22). Plasma ACTH played a mediation role in the estimated effect between 1-mg DST and DHEAS and contralateral diameter.

AIs are commonly discovered during abdominal imaging. Although these incidentally discovered adrenal masses are mostly benign and asymptomatic, MACS account for approximately 12-30% of Ais (4, 7, 23). Several studies had suggested that MACS may be associated with an increased risk for bone fracture (24, 25), cardiovascular disease and mortality. However, a recent study also found no elevated risk of hypertension, diabetes, and dyslipidemia in patients with MACS (1-mg DST: 1.8-5.0ug/dL) compared with NFAT (12). Although, dexamethasone is a potent synthetic glucocorticoid, which could suppress corticotropin secretion in normal subjects (26), the cutoff value for the serum cortisol level after 1-mg DST to make a diagnosis of mild autonomous cortisol excess is controversial. A level of more than 1.8 ug/dL has high sensitivity (95 to 100%) but low specificity (60 to 80%), whereas a level of more than 5.0ug/dL has lower sensitivity (86%) but higher specificity (92 to 97%) (27).

DHEAS could reflect a chronically suppressed HPA axis due to the relatively stable levels throughout the day (28). Multiple studies have assessed the diagnostic accuracy of DHEAS in confirmed MACS (29–31). A recent study by Liu et al. suggested that a single measurement of DHEAS is beneficial for identifying MACS (30); even circulating DHEA(S) levels decline with age and are associated with gender (32). However, they used a level of more than 1.8ug/dL of low-dose DST (LDDST) to diagnose MACS, which may overestimate the findings. The reason why the differences in DHEAS in our study were not more obvious may be related to the various diagnostic criteria and individuals included.

Adrenal tumors with overt Cushing’s syndrome presented with atrophy of the remaining contralateral adrenal gland due to suppressed ACTH production. Based on this pathophysiological phenomenon, several studies regarding the association between radiological characteristics and endocrine hormones have been made. Kong et al. found a negative linear correlation between limb width of the contralateral adrenal gland and cortisol levels after DST in patients with unilateral adrenal adenomas (18). Another study showed the significance of the volume of adrenal adenomas and contralateral adrenal glands associated with cortisol secretion (19). In our study, we found that patients with MACS had thinner contralateral adrenal compared with NFAT. Plasma ACTH was significantly associated with a decreased contralateral diameter in total patients, although there was no such association in the subgroup analysis of the MACS group. This may be related to the smaller number of patients in the MACS group, and the exact duration time of the HPA axis changes is unknown due to the nature of retrospective cross-sectional studies. Prospective studies are needed to evaluate how long it takes for changes in the HPA axis to have an effect on adrenal limb width.

Our study has several strengths. First, we comprehensively demonstrated the radiological and clinical characteristics of patients with MACS. The difference between MACS and NFAT indicated that clinical follow-up is essential. Second, we performed the causal mediation analysis to further investigate the association between radiological features and the HPA axis. This analysis was able to provide quantitative evidence supporting the role of ACTH as a mediator in the association between mild autonomous cortisol secretion and DHEAS and the contralateral adrenal gland. Third, this study is based on a relatively larger cohort in data regarding the association between radiological characteristics and hormone functionality. Large-scale analyses may give a high level of statistical power to detect effects.

This study also has several limitations. First, the study was cross-sectional and could not evaluate the association between radiologic features and the prognosis of MACS. Second, the cardiovascular morbidity in our cohort was not significantly different between two groups. Though this was in accordance with another recent large cohort, given the aim of our study, the fact that we did not include patients with bilateral tumors who had higher risks of morbidity may also account for this. Third, different types of CT protocols (abdomen or adrenal) were included in the study, which may affect diameter measurements. However, the difference could be minimal since the scan slice interval of each CT protocol was similar between 3 and 5 mm. The measurement is taken as the maximum width of the adrenal gland in the transverse plane rather than continuous measurements along the longitudinal axis. Therefore, the difference in slice interval may have a minimal impact on the measurement results.

In conclusion, our study demonstrated that patients with MACS had a higher tumor diameter, thinner contralateral adrenal diameter, and disturbed HPA axis. ACTH may partially be involved in mediating the mild autonomous cortisol secretion to the DHEAS and contralateral adrenal gland. The association between radiological features and the HPA axis indicated prospective studies are needed to assess risks in this population.

## Data availability statement

The raw data supporting the conclusions of this article will be made available by the authors, without undue reservation.

## Ethics statement

The studies involving human participants were reviewed and approved by Ruijin Hospital, Shanghai Jiao Tong University School of Medicine. The patients/participants provided their written informed consent to participate in this study.

## Author contributions

WW conceptualized the study. TS, WZ, YJ, and XZ designed the study. LJ and CZ collected the data. HZ and LW performed the analysis and drafted the article. All authors contributed to critical revisions and final approval of the article.

## Supplementary material

Supplementary Figure 1 The schematic diagram of the brief evaluation process of patients with adrenal incidentaloma in our center.

Supplementary Figure 2 The sensitive analysis of causal mediation analysis among radiological characteristics, 1-mg DST and endocrine assessments. **(A)** mediation linkages between ACTH and 1-mg DST contributed DHEAS. **(B)** mediation linkages between contralateral diameter and 1-mg DST contributed DHEAS. **(C)** mediation linkages between ACTH and 1-mg DST contributed contralateral diameter. **(D)** mediation linkages between contralateral diameter and ACTH contributed DHEAS. *P < 0.05, **P < 0.01, and ***P < 0.001.

## References

[1] Young WF Jr. Clinical practice. The incidentally discovered adrenal mass. N Engl J Med (2007) 356(6):601–10. doi: 10.1056/NEJMcp065470 17287480

[2] Davenport C Liew A Doherty B Win HH Misran H Hanna S. The prevalence of adrenal incidentaloma in routine clinical practice. Endocrine (2011) 40(1):80–3. doi: 10.1007/s12020-011-9445-6 21547511

[3] Kloos RT Gross MD Francis IR Korobkin M Shapiro B. Incidentally discovered adrenal masses. Endocr Rev (1995) 16(4):460–84. doi: 10.1210/edrv-16-4-460 8521790

[4] Fassnacht M Arlt W Bancos I Dralle H Newell-Price J Sahdev A. Management of adrenal incidentalomas: European Society of Endocrinology Clinical Practice Guideline in collaboration with the European Network for the Study of Adrenal Tumors. Eur J Endocrinol (2016) 175(2):G1–G34. doi: 10.1530/EJE-16-0467 27390021

[5] Fassnacht M Tsagarakis S Terzolo M Tabarin A Sahdev A Newell-Price J. European Society of Endocrinology Clinical Practice Guidelines on the management of adrenal incidentalomas, in collaboration with the European Network for the Study of Adrenal Tumors. Eur J Endocrinol (2023). doi: 10.1093/ejendo/lvad066 37318239

[6] Debono M Bradburn M Bull M Harrison B Ross RJ Newell-Price J. Cortisol as a marker for increased mortality in patients with incidental adrenocortical adenomas. J Clin Endocrinol Metab (2014) 99(12):4462–70. doi: 10.1210/jc.2014-3007 PMC4255126 25238207

[7] Di Dalmazi G Vicennati V Garelli S Casadio E Rinaldi E Giampalma E. Cardiovascular events and mortality in patients with adrenal incidentalomas that are either non-secreting or associated with intermediate phenotype or subclinical Cushing's syndrome: a 15-year retrospective study. Lancet Diabetes Endocrinol (2014) 2(5):396–405. doi: 10.1016/S2213-8587(13)70211-0 24795253

[8] Morelli V Palmieri S Lania A Tresoldi A Corbetta S Cairoli E. Cardiovascular events in patients with mild autonomous cortisol secretion: analysis with artificial neural networks. Eur J Endocrinol (2017) 177(1):73–83. doi: 10.1530/EJE-17-0047 28468767

[9] Patrova J Kjellman M Wahrenberg H Falhammar H. Increased mortality in patients with adrenal incidentalomas and autonomous cortisol secretion: a 13-year retrospective study from one center. Endocrine (2017) 58(2):267–75. doi: 10.1007/s12020-017-1400-8 28887710

[10] Sbardella E Minnetti M D'Aluisio D Rizza L Di Giorgio MR Vinci F. Cardiovascular features of possible autonomous cortisol secretion in patients with adrenal incidentalomas. Eur J Endocrinol (2018) 178(5):501–11. doi: 10.1530/EJE-17-0986 29510982

[11] Kjellbom A Lindgren O Puvaneswaralingam S Londahl M Olsen H. Association between mortality and levels of autonomous cortisol secretion by adrenal incidentalomas : A cohort study. Ann Intern Med (2021) 174(8):1041–9. doi: 10.7326/M20-7946 34029490

[12] Prete A Subramanian A Bancos I Chortis V Tsagarakis S Lang K. Cardiometabolic disease burden and steroid excretion in benign adrenal tumors : A cross-sectional multicenter study. Ann Intern Med (2022) 175(3):325–34. doi: 10.7326/M21-1737 34978855

[13] Yener S Secil M Demir O Ozgen Saydam B Yorukoglu K. Chemical shift magnetic resonance imaging could predict subclinical cortisol production from an incidentally discovered adrenal mass. Clin Endocrinol (Oxf) (2018) 88(6):779–86. doi: 10.1111/cen.13587 29498083

[14] Mosconi C Vicennati V Papadopoulos D Dalmazi GD Morselli-Labate AM Golfieri R. Can imaging predict subclinical cortisol secretion in patients with adrenal adenomas? A CT predictive score. AJR Am J Roentgenol (2017) 209(1):122–9. doi: 10.2214/AJR.16.16965 28402131

[15] Olsen H Nordenstrom E Bergenfelz A Nyman U Valdemarsson S Palmqvist E. Subclinical hypercortisolism and CT appearance in adrenal incidentalomas: a multicenter study from Southern Sweden. Endocrine (2012) 42(1):164–73. doi: 10.1007/s12020-012-9622-2 22350586

[16] Golden SH Malhotra S Wand GS Brancati FL Ford D Horton K. Adrenal gland volume and dexamethasone-suppressed cortisol correlate with total daily salivary cortisol in African-American women. J Clin Endocrinol Metab (2007) 92(4):1358–63. doi: 10.1210/jc.2006-2674 17284636

[17] Huayllas MKP Sirineni GK Smith LM Gallagher JC Singh RJ Netzel BC. Correlation between size and function of unilateral and bilateral adrenocortical nodules: an observational study. AJR Am J Roentgenol (2020) 214(4):800–7. doi: 10.2214/AJR.19.21753 32069079

[18] Kong SH Kim JH Shin CS. Contralateral adrenal thinning as a distinctive feature of mild autonomous cortisol excess of the adrenal tumors. Eur J Endocrinol (2020) 183(3):325–33. doi: 10.1530/EJE-20-0301 32717717

[19] Olmos R Mertens N Vaidya A Uslar T Fernandez P Guarda FJ. Discriminative capacity of volumetry by CT scan to identify autonomous cortisol secretion in incidental adrenal adenomas. J Clin Endocrinol Metab (2022) 107 (5) :e1946-e53. doi: 10.1210/clinem/dgac005 PMC9272424 35020922

[20] Vincent JM Morrison ID Armstrong P Reznek RH. The size of normal adrenal glands on computed tomography. Clin Radiol (1994) 49(7):453–5. doi: 10.1016/S0009-9260(05)81739-8 8088036

[21] Wang S Li M Lin H Wang G Xu Y Zhao X. Amino acids, microbiota-related metabolites, and the risk of incident diabetes among normoglycemic Chinese adults: Findings from the 4C study. Cell Rep Med (2022) 3 (9): 100727. doi: 10.1016/j.xcrm.2022.100727 35998626 PMC9512668

[22] Xing Y Edwards MA Ahlem C Kennedy M Cohen A Gomez-Sanchez CE. The effects of ACTH on steroid metabolomic profiles in human adrenal cells. J Endocrinol (2011) 209(3):327–35. doi: 10.1530/JOE-10-0493 PMC3774117 21429963

[23] Mantero F Terzolo M Arnaldi G Osella G Masini AM Ali A. A survey on adrenal incidentaloma in Italy. Study Group on Adrenal Tumors of the Italian Society of Endocrinology. J Clin Endocrinol Metab (2000) 85(2):637–44. doi: 10.1210/jc.85.2.637 10690869

[24] Morelli V Eller-Vainicher C Palmieri S Cairoli E Salcuni AS Scillitani A. Prediction of vertebral fractures in patients with monolateral adrenal incidentalomas. J Clin Endocrinol Metab (2016) 101(7):2768–75. doi: 10.1210/jc.2016-1423 27144939

[25] Salcuni AS Morelli V Eller Vainicher C Palmieri S Cairoli E Spada A. Adrenalectomy reduces the risk of vertebral fractures in patients with monolateral adrenal incidentalomas and subclinical hypercortisolism. Eur J Endocrinol (2016) 174(3):261–9. doi: 10.1530/EJE-15-0977 26630908

[26] Nugent CA Macdiarmid WD Nelson AR Tyler FH. Rate of adrenal cortisol production in response to maximal stimulation with ACTH. J Clin Endocrinol Metab (1963) 23:684–93. doi: 10.1210/jcem-23-7-684 13939197

[27] Kebebew E. Adrenal incidentaloma. N Engl J Med (2021) 384(16):1542–51. doi: 10.1056/NEJMcp2031112 33882207

[28] Klinge CM Clark BJ Prough RA. Dehydroepiandrosterone research: past, current, and future. Vitam Horm (2018) 108:1–28. doi: 10.1016/bs.vh.2018.02.002 30029723

[29] Dennedy MC Annamalai AK Prankerd-Smith O Freeman N Vengopal K Graggaber J. Low DHEAS: A sensitive and specific test for the detection of subclinical hypercortisolism in adrenal incidentalomas. J Clin Endocrinol Metab (2017) 102(3):786–92. doi: 10.1210/jc.2016-2718 27797672

[30] Liu MS Lou Y Chen H Wang YJ Zhang ZW Li P. Performance of DHEAS as a screening test for autonomous cortisol secretion in adrenal incidentalomas: A prospective study. J Clin Endocrinol Metab (2022) 107 (5) :e1789-e96. doi: 10.1210/clinem/dgac072 35137142

[31] Yener S Yilmaz H Demir T Secil M Comlekci A. DHEAS for the prediction of subclinical Cushing's syndrome: perplexing or advantageous? Endocrine (2015) 48(2):669–76. doi: 10.1007/s12020-014-0387-7 25146553

[32] Labrie F Luu-The V Belanger A Lin SX Simard J Pelletier G. Is dehydroepiandrosterone a hormone? J Endocrinol (2005) 187(2):169–96. doi: 10.1677/joe.1.06264 16293766

