# Supplementary material for: Association between contralateral adrenal and hypothalamus-pituitary-adrenal axis in benign adrenocortical tumors
Source: Front Endocrinol (Lausanne). 2023 Jul 25;14:1199875. doi: 10.3389/fendo.2023.1199875 (PMC10407553; doi:10.3389/fendo.2023.1199875)
Supplement: Supplementary file 1 [file Table_1.docx]

**Supplementary Table 1. Clinical and radiological characteristics of patients according to gender.**

| Characteristic | Male | | | Female | | |
| --- | --- | --- | --- | --- | --- | --- |
|  | NFAT (n=178) | MACS (n=49) | P value | NFAT (n=174) | MACS (n=93) | P value |
| Gender (male/female) | 178/0 | 49/0 | - | 0/174 | 0/93 | - |
| Age (years) | 52 (42, 59) | 58 (49, 65) | 0.004 | 57 (49, 63) | 57 (50, 65) | 0.356 |
| BMI (Kg/㎡) | 25.48 (23.68, 27.38) | 24.77 (21.70, 27.53) | 0.275 | 24.35 (22.19, 26.31) | 24.10 (21.79, 27.70) | 0.229 |
| Serum cortisol (8am) (ug/dl) | 11.21 (8.64, 14.48) | 13.06 (10.02, 17.06) | 0.009 | 11.42 (8.54, 14.37) | 12.13 (9.79, 15.22) | 0.138 |
| Serum cortisol (0am) (ug/dl) | 2.85 (1.88, 3.97) | 4.14 (2.49, 5.97) | <0.001 | 2.91 (1.93, 5.21) | 4.03 (3.10, 5.30) | 0.036 |
| ACTH (pg/ml) | 27.23 (19.83, 44.4) | 21.30 (14.99, 35.17) | 0.005 | 24.14 (14.75, 33.72) | 16.59 (13.37, 25.48) | <0.001 |
| 24h-UFC (ug/24h) | 102.24 (75.54, 127.09) | 105.77 (86.66, 148.52) | 0.272 | 74.62 (58.95, 95.47) | 82.57 (64.06, 112.28) | 0.047 |
| Cortisol after 1mg DST (ug/dl) | 1.05 (0.80, 1.40) | 2.50 (2.02, 3.03) | <0.001 | 1.10 (0.87, 1.34) | 2.30 (2.00, 2.85) | <0.001 |
| DHEAS (ug/dl) | 190.2 (120.3, 283.4) | 117.5 (83.2, 184.0) | <0.001 | 100.8 (62.7, 155.5) | 77.9 (48.9, 120.3) | 0.006 |
| SHBG (nmol/L) | 25.6 (19.7, 36.2) | 33.8 (21.7, 49.0) | 0.004 | 41.2 (29.3, 61.3) | 39.5 (27.4, 58.3) | 0.617 |
| AD (ng/mL) | 1.43 (1.06, 1.77) | 1.27 (1.03, 1.53) | 0.124 | 1.06 (0.73, 1.36) | 1.04 (0.81, 1.26) | 0.640 |
| T (ng/mL) | 4.05 (3.29, 5.12) | 4.32 (3.05, 5.51) | 0.720 | 0.23 (0.16, 0.33) | 0.22 (0.14, 0.31) | 0.180 |
| FT (pg/ml) | 8.44 (6.83, 10.46) | 7.66 (6.52, 8.81) | 0.042 | 1.33 (0.95, 1.77) | 1.25 (0.89, 1.66) | 0.423 |
| DHT (pg/ml) | 226.89 (142.05, 286.56) | 228.99 (123.41, 324.41) | 0.579 | 32.51 (21.60, 47.93) | 24.36 (17.51, 39.49) | 0.282 |
| CT attenuation value (HU) | 11.6 (-1.9, 20.6) | 9.7 (2.6, 21.8) | 0.796 | 9.3 (-1.2, 19.5) | 11.3 (-2.1, 21.7) | 0.628 |
| Tumor diameter (mm) | 20.2 (15.7, 26.9) | 25.8 (19.5, 32.8) | <0.001 | 20.5 (15.6, 25.1) | 21.1 (16.6, 29.0) | 0.051 |
| Contralateral diameter (mm) | 3.8 (3.1, 4.2) | 3.4 (3.0, 3.8) | 0.023 | 3.4 (2.9, 3.9) | 3.1 (2.7, 3.5) | 0.083 |
| Diabetes (%) | 25.1% | 22.9% | 0.938 | 18.8% | 15.1% | 0.094 |
| Hypertension (%) | 57.3% | 61.2% | 0.786 | 52.0% | 62.6% | 0.131 |
| Dyslipidemia (%) | 14.9% | 20.8% | 0.616 | 12.7% | 11.2% | 0.941 |

Data were medians (interquartile ranges) for continuous variables, or proportions for categorical variables.
