# Supplementary material for: Association between contralateral adrenal and hypothalamus-pituitary-adrenal axis in benign adrenocortical tumors
Source: Front Endocrinol (Lausanne). 2023 Jul 25;14:1199875. doi: 10.3389/fendo.2023.1199875 (PMC10407553; doi:10.3389/fendo.2023.1199875)
Supplement: Supplementary file 2 [file Table_2.docx]

**Supplementary Table 2. Logistic regression analysis of decreased DHEAS in patients with MACS.**

|  | Decreased DHEAS level | | | |
| --- | --- | --- | --- | --- |
|  | Univariable | | Multivariable | |
|  | OR (95% CI) | P value | OR (95% CI) | P value |
| ACTH | 0.948 (0.913-0.985) | 0.007 | 0.937 (0.888-0.988) | 0.016 |
| Serum cortisol (8am) | 0.941 (0.864-1.025) | 0.162 | - | - |
| Serum cortisol (0am) | 0.984 (0.880-1.099) | 0.771 | - | - |
| Cortisol after 1-mg DST | 0.655 (0.423-1.015) | 0.059 | 0.751 (0.420-1.342) | 0.333 |
| Tumor diameter | 1.416 (0.916-2.188) | 0.117 | - | - |
| Contralateral diameter | 0.508 (0.262-0.986) | 0.045 | 0.472 (0.196-1.139) | 0.095 |

Odds ratio (OR) and 95% confidence interval (CI) was evaluated using logistic regression models, multivariable model was adjusted for age and gender.
