# Supplementary material for: Association between contralateral adrenal and hypothalamus-pituitary-adrenal axis in benign adrenocortical tumors
Source: Front Endocrinol (Lausanne). 2023 Jul 25;14:1199875. doi: 10.3389/fendo.2023.1199875 (PMC10407553; doi:10.3389/fendo.2023.1199875)
Supplement: Supplementary file 3 [file Table_3.docx]

**Supplementary Table 3. Logistic regression analysis of decreased contralateral diameter in patients with MACS.**

|  | Decreased contralateral diameter | | | |
| --- | --- | --- | --- | --- |
|  | Univariable | | Multivariable | |
|  | OR (95% CI) | P value | OR (95% CI) | P value |
| ACTH | 0.978 (0.948-1.008) | 0.146 | - | - |
| Serum cortisol (8am) | 0.928 (0.852-1.010) | 0.084 | 0.937 (0.853-1.031) | 0.182 |
| Serum cortisol (0am) | 0.890 (0.785-1.010) | 0.072 | 0.919 (0.805-1.049) | 0.211 |
| Cortisol after 1mg DST | 1.250 (0.786-1.990) | 0.346 | - | - |
| Tumor diameter | 0.800 (0.572-1.119) | 0.193 | - | - |

Odds ratio (OR) and 95% confidence interval (CI) was evaluated using logistic regression models, multivariable model was adjusted for age and gender.
