# Supplementary material for: Association between contralateral adrenal and hypothalamus-pituitary-adrenal axis in benign adrenocortical tumors
Source: Front Endocrinol (Lausanne). 2023 Jul 25;14:1199875. doi: 10.3389/fendo.2023.1199875 (PMC10407553; doi:10.3389/fendo.2023.1199875)
Supplement: Supplementary file 4 [file Table_4.docx]

**Supplementary Table 4. Association between adenoma diameter and plasma ACTH**

| Mediator | ACME | ADE | Total effect | Prop. mediated |
| --- | --- | --- | --- | --- |
| Serum cortisol (8am) | -0.030 (-0.106, 0.010) | -0.197 (-0.578, 0.010) | -0.227 (-0.629, 0)* | 0.131 (-0.146, 0.710) |
| Serum cortisol (0am) | 0.002 (-0.016, 0.020) | -0.228 (-0.627, 0)* | -0.227 (-0.623, 0)* | -0.007 (-0.171, 0.150) |
| Cortisol after 1-mg DST | -0.055 (-0.118, -0.020)*** | -0.172 (-0.545, 0.040) | -0.227 (-0.624, 0)* | 0.242 (0.029, 1.590)* |

ACME: average casual mediation effect, ADE: average direct effect, Prop. Mediated: the proportion of mediation effect. Data were estimate with 95% confidence interval (CI). *P < 0.05, **P < 0.01, ***P < 0.001
