# Supplementary figures and images for: Association between contralateral adrenal and hypothalamus-pituitary-adrenal axis in benign adrenocortical tumors
Source: Front Endocrinol (Lausanne). 2023 Jul 25;14:1199875. doi: 10.3389/fendo.2023.1199875 (PMC10407553; doi:10.3389/fendo.2023.1199875)

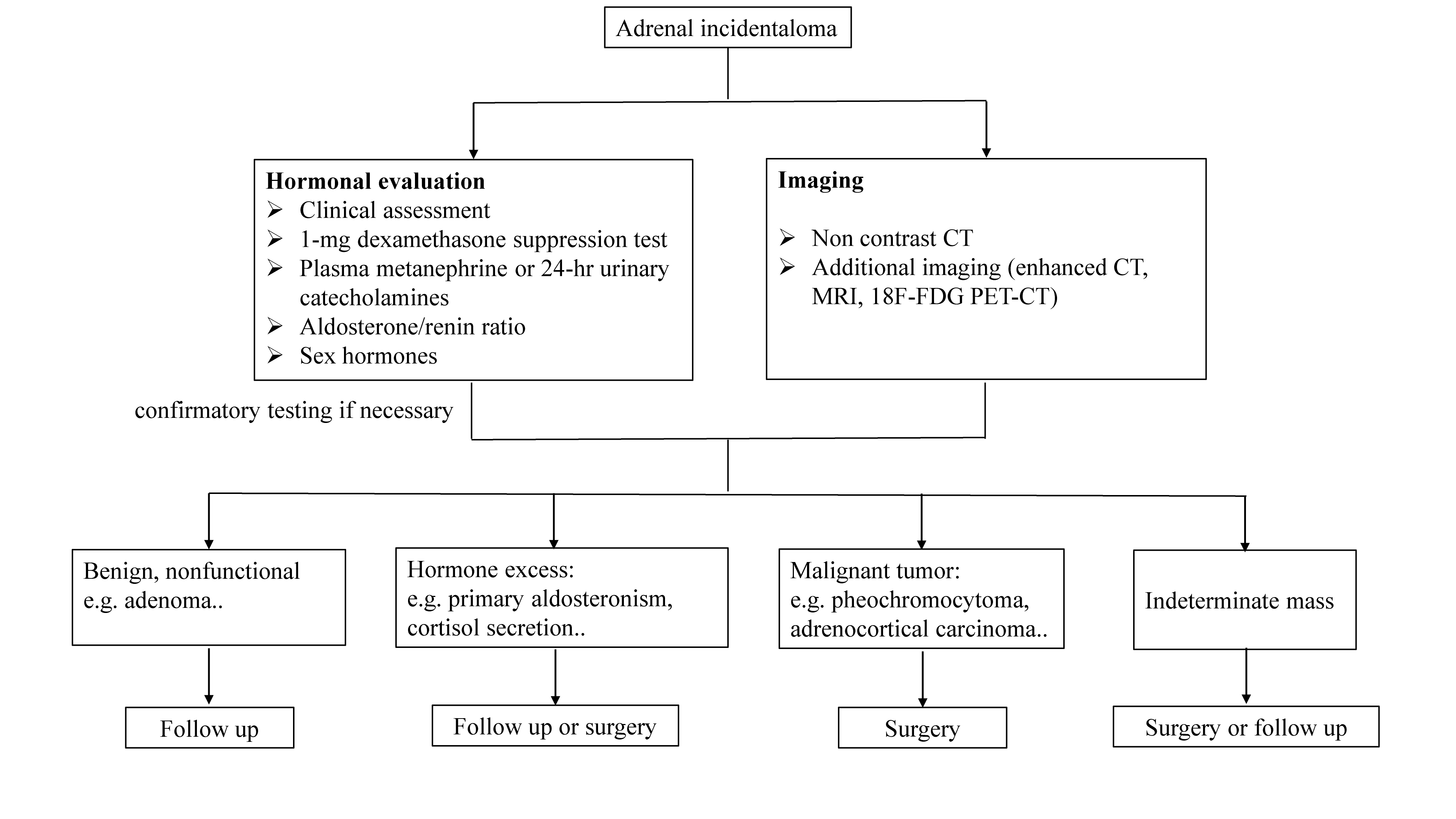

Supplement: Supplementary Figure 1 — The schematic diagram of the brief evaluation process of patients with adrenal incidentaloma in our center. [file Image_1.tif]

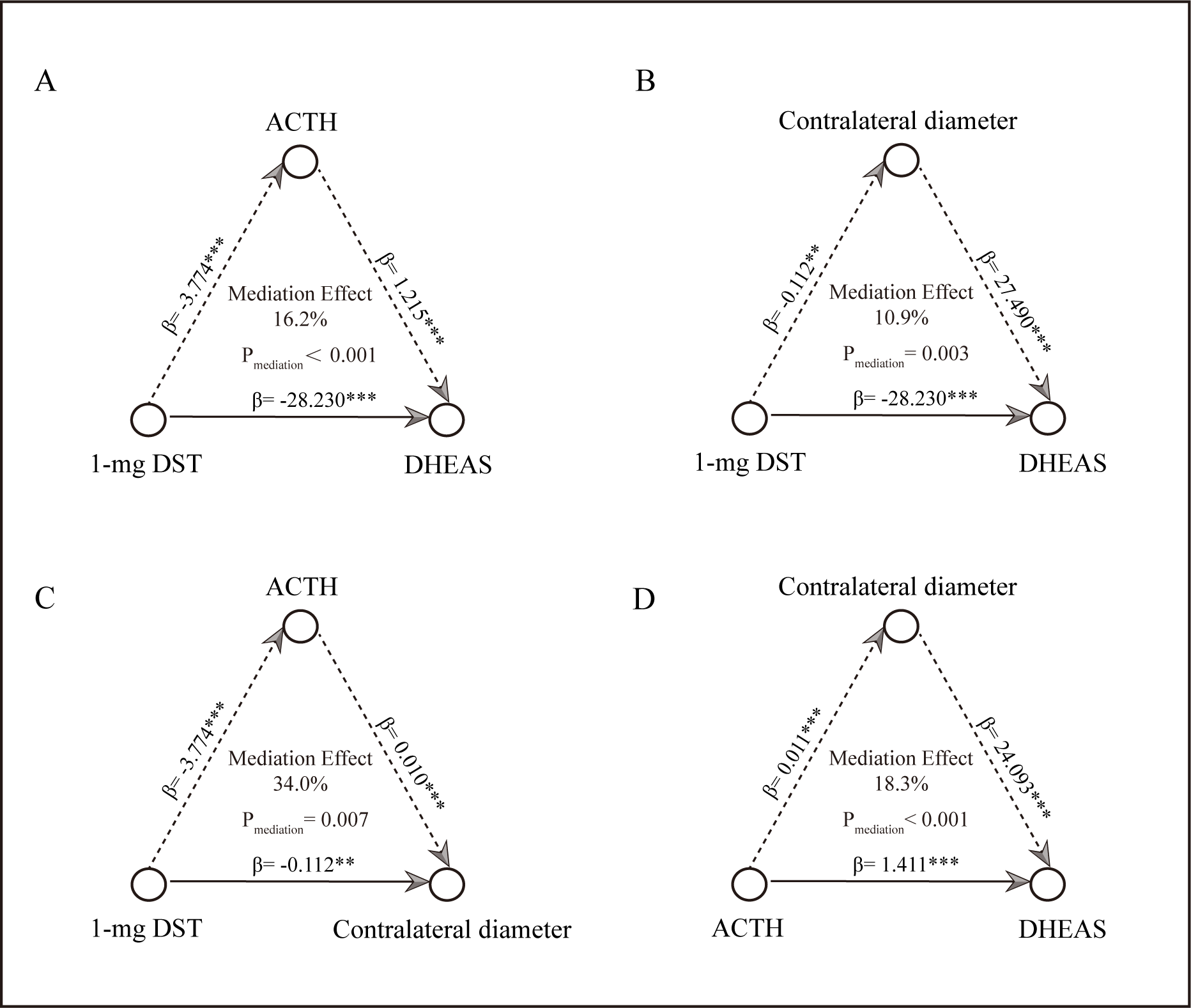

Supplement: Supplementary Figure 2 — The sensitive analysis of causal mediation analysis among radiological characteristics, 1-mg DST and endocrine assessments. (A) mediation linkages between ACTH and 1-mg DST contributed DHEAS. (B) mediation linkages between contralateral diameter and 1-mg DST contributed DHEAS. (C) mediation linkages between ACTH and 1-mg DST contributed contralateral diameter. (D) mediation linkages between contralateral diameter and ACTH contributed DHEAS. *P < 0.05, **P < 0.01, and ***P < 0.001. [file Image_2.tif]
